# The Safety Profile of Inclisiran in Patients with Dyslipidemia: A Systematic Review and Meta-Analysis

**DOI:** 10.3390/healthcare13020141

**Published:** 2025-01-13

**Authors:** Maisha Maliha, Vikyath Satish, Sriram Sunil Kumar, Kuan Yu Chi, Nishat Shama, Amrin Kharawala, Gustavo Duarte, Weijia Li, Sutopu Purkayastha, Shaunak Mangeshkar, Pawel Borkowski, Eleonora Gashi, Supreeti Behuria

**Affiliations:** 1 Jacobi Medical Center/New York City Health and Hospitals Corporation, Department of Medicine, 1400 Pelham Pkwy S, Bronx, New York, NY 10461, USA; 2 Albert Einstein College of Medicine, Department of Medicine, 1300 Morris Park Ave, Bronx, New York, NY 10461, USA; 3 Henry Ford Hospital, Department of Medicine, 2799 W Grand Blvd, Detroit, MI 48202, USA; 4 Cleveland Clinic Florida, Department of Cardiology, 3100 Weston Rd, Weston, FL 33331, USA; 5 Advent Health, Department of Cardiology, 601 E Rollins St, Orlando, FL 32803, USA; 6 Northwell Health, Department of Cardiology, 501 Seaview Avenue, Suite 200, Staten Island, NY 10305, USA

**Keywords:** inclisiran, safety, LDL, lipid

## Abstract

Introduction: Inclisiran is a novel drug that employs ribonucleic acid (RNA) interference to lower the levels of the proprotein convertase subtilisin/kexin type 9 (PCSK9) protein. It has demonstrated a significant reduction in LDL cholesterol levels compared to a placebo. We aim to comprehensively evaluate the safety of using Inclisiran in patients with dyslipidemia and ASCVD or an ASCVD risk equivalent. Methods: Four electronic databases, namely, Pubmed/MEDLINE, Web of Science, Embase, and ClinicalTrials.gov, were searched from inception to June 2024 to identify relevant randomized controlled trials (RCTs) comparing safety profiles of Inclisiran and the control group. The outcomes investigated were all-cause mortality, major adverse cardiovascular events (MACEs), injection-site adverse events, new-onset or worsening type 2 diabetes mellitus (T2DM), and nasopharyngitis. The effect estimates of outcomes were assessed using the risk ratio (RR) with a 95% confidence interval (CI). Random-effects meta-analysis was conducted using the restricted maximum likelihood method. Subgroup analysis was performed based on different dosing regimens. Results: The study included 7 RCTs, enrolling 4790 patients (age 63.8 ± 9.7 years, 33.2% females) who received Inclisiran. Compared to the control group, Inclisiran use did not yield a significant effect on all-cause mortality (RR, 0.92; 95% CI, 0.54 to 1.54; I^2^ = 0%), MACEs (RR, 0.98; 95% CI, 0.82 to 1.17; I^2^ = 0%), nasopharyngitis (RR, 1.10; 95% CI, 0.83 to 1.45; I^2^ = 0%), and T2DM (RR, 1.02; 95% CI, 0.85 to 1.21; I^2^ = 0%). However, Inclisiran use demonstrated a significant increase in injection-site adverse events (RR, 6.50; 95% CI, 3.20 to 13.20; I^2^ = 29%). Conclusions: Inclisiran use significantly increased injection-site reactions, with no increase in mortality, T2DM, or nasopharyngitis. It demonstrates a generally favorable safety profile, making it a promising option for lipid management in individuals at high cardiovascular risk, such as those with ASCVD or equivalent conditions. While it effectively improves dyslipidemia, decision-makers should be aware of an increased incidence of injection-site reactions, which, though typically mild, warrant consideration in clinical practice. Further trials are required to assess the safety of Inclisiran, particularly the association of the severity of injection-site adverse events over longer treatment durations.

## 1. Introduction

Atherosclerotic cardiovascular disease (ASCVD) is the leading cause of death in the world, impacting over 500 million individuals globally and resulting in approximately 19 million deaths annually [1,2,3]. In the United States, it affects around 26 million people, leading to 2 million hospitalizations and 400,000 deaths per year [4,5]. The role of elevated cholesterol levels, especially low-density lipoprotein (LDL-C), has been studied in multifaceted clinical trials and shown to be a major contributor to ASCVD [6,7]. High-intensity statins are the first line of defense for the prevention and treatment of ASCVD in high-risk individuals [6,8]. Ezetimibe further reduces LDL-C levels by up to 24% and serves as an adjunct to statins in individuals without significant responses to maximally tolerated doses of statins [6,9]. However, studies have shown that despite the availability of statins and ezetimibe, a large number of high-risk patients fail to achieve the target LDL due to prescribing patterns and non-adherence [10,11,12].

The introduction of medications that target proprotein convertase subtilisin–kexin type 9 (PCSK9) offers a novel approach to lipid-lowering therapy [13,14]. PCSK9 is a protein produced by the liver that binds LDL receptors to its surface [13,14]. Monoclonal antibodies against PCKS9 significantly decrease LDL levels by 65%, regardless of their combination with other lipid-lowering therapies, along with a decrease in major adverse cardiac events (MACEs) [15]. A meta-analysis of these studies demonstrated no significant differences in treatment-emergent adverse events (TEAEs) between PCSK9 antibodies and the placebo [11].

A new frontier in lipid-lowering therapies has been opened with the introduction of Inclisiran, favored for its less frequent dosing regimen [16]. Inclisiran uses small interfering RNA (siRNA) to reduce LDL levels. The siRNA binds to the messenger RNA (mRNA) that codes for PCSK9 along with an RNA-inducing silencing complex (RISC) [16,17]. This combination activates RNAase, which cleaves the mRNA, preventing the production of PCSK9 and thereby decreasing serum LDL levels [16,17]. Recent clinical trials in patients with dyslipidemias and elevated ASCVD risk showed that Inclisiran, dosed once every three to six months, causes significant reductions in circulating LDL [14,18,19,20,21].

This systematic review and meta-analysis aims to evaluate the safety profile of Inclisiran in patients with dyslipidemia and ASCVD or ASCVD risk equivalents, as reported in the current literature. While Inclisiran has demonstrated significant efficacy in lowering LDL-C levels and reducing ASCVD risk, understanding its safety profile relative to its therapeutic benefits is essential for its optimal integration into clinical practice. Evaluating safety is particularly important given the chronic nature of lipid-lowering therapies and the potential for long-term adverse effects, which could impact patient adherence and outcomes. By comprehensively assessing safety data alongside efficacy outcomes, this review seeks to provide clinicians with actionable insights into the risk–benefit profile of Inclisiran, thereby enhancing decision-making in cardiovascular care and improving patient management strategies.

Summary: Inclisiran, a drug that lowers PCSK9 levels, was evaluated in seven RCTs involving 4790 patients with dyslipidemia and ASCVD risk, showing no significant effect on all-cause mortality, MACEs, nasopharyngitis, or T2DM. However, it was associated with a significant increase in injection-site adverse events, suggesting a generally favorable safety profile for lipid management in high-cardiovascular-risk patients.

## 2. Methods

### 2.1. Database Search and Study Eligibility

The electronic databases PubMed/MEDLINE, Embase, Web of Science, and ClinicalTrails. Gov were reviewed from inception until June 2024. Systematic literature searches were conducted using a combination of MeSH terms, namely, “Dyslipidemia” or “ASCVD” or “Hyperlipidemia” or “LDL” and “Inclisiran” and “Clinical trials” or “Clinical studies”, to identify human studies in which Inclisiran was used in patients with dyslipidemia and ASCVD or risk equivalents.

Randomized controlled trials (RCTs) were screened for eligibility based on adults aged ≥18 years who were on Inclisiran for dyslipidemia and ASCVD or ASCVD risk equivalents. The term ASCVD risk equivalent refers to individuals without a known history of ASCVD but who either have T2DM, heterozygous familial hypercholesterolemia, or a 10-year cardiovascular risk greater than 20% based on the Framingham Risk Score or an equivalent assessment [22,23]. RCTs without an appropriate control group for Inclisiran administration, extensions of previous trials and crossover portion of RCTs, retrospective studies, cross-sectional or prospective observational studies, review articles, case reports, case series, editorials, and non-human studies were excluded from this meta-analysis.

The outcomes of interest were (i) all-cause mortality; (ii) a composite of major adverse cardiovascular events (MACEs), which included non-adjudicated exploratory cardiovascular events classified in the Medical Dictionary for Regulatory Activities such as cardiac death, any signs or symptoms of cardiac arrest, nonfatal myocardial infarction (MI), and nonfatal stroke; [14,18,19,20,21,24] (iii) injection-site adverse events, which include injection-site erythema, pain, hypersensitivity, pruritus, rash, and thrombophlebitis; [14,18,19,20,21,24] (iv) the new onset or worsening of T2DM; and (v) nasopharyngitis.

This study used the Preferred Reporting Items for Systematic Reviews and Meta-Analyses (PRISMA) guidelines [25]. Covidence software, a web-based collaboration platform, was used to streamline the screening process [26]. The study design and protocol are registered with the PROSPERO International Prospective Register of Systematic Reviews (registration number CRD42024537358).

### 2.2. Study Selection and Data Extraction

**Figure 1:**
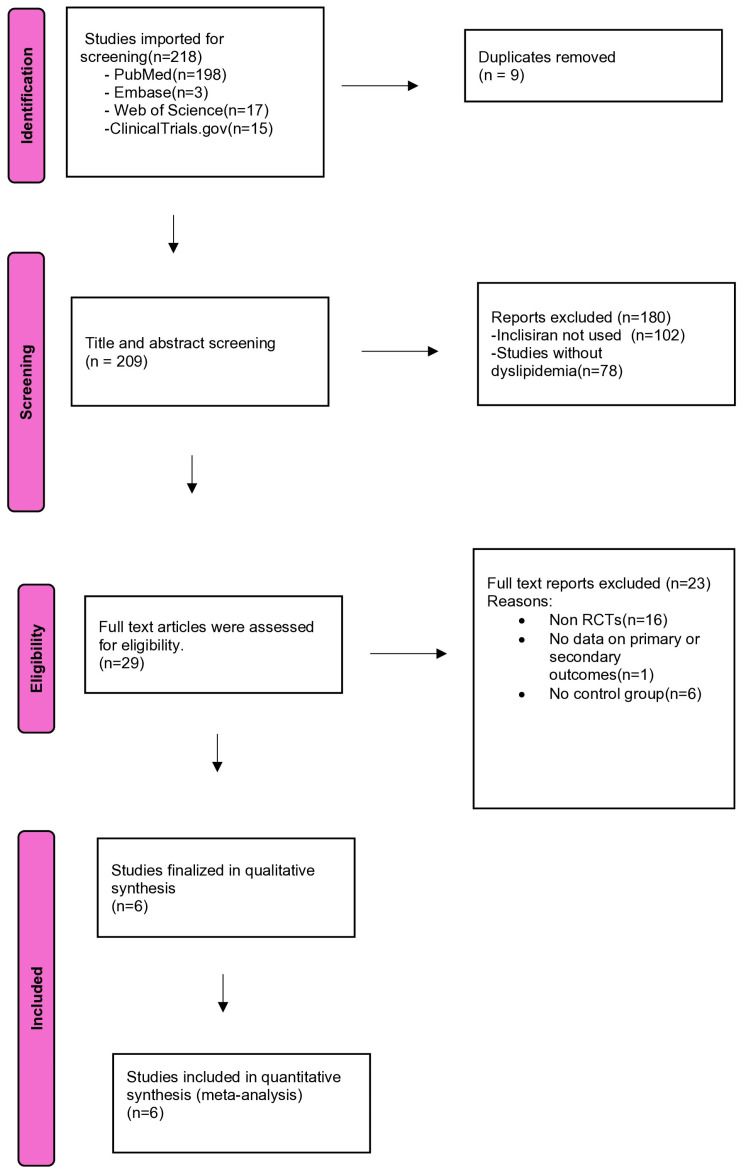
A PRISMA flow diagram. Summary: The PRISMA flow diagram summarizes the study selection process, where 218 studies were screened, 6 were included in the final qualitative and quantitative synthesis, and the rest were excluded due to duplication, irrelevant interventions, or a lack of eligibility criteria.

Two independent reviewers (M.M. and V.S.) participated in the initial screening process through abstract and title review. Additionally, review articles, retrospective studies, and other irrelevant studies were excluded. Conflicts between those two reviewers were evaluated by two other different team members (S.K. and K.Y.). The full-text screening was performed over two rounds (twice) and undertaken by four reviewers (M.M., V.S., S.K., and K.Y.), with two in each round. Conflicts between the four reviewers were resolved by reaching a consensus among the authors. The PRISMA flow diagram detailing the process is illustrated in Figure 1.

### 2.3. Statistical Analysis

**Figure 2:**
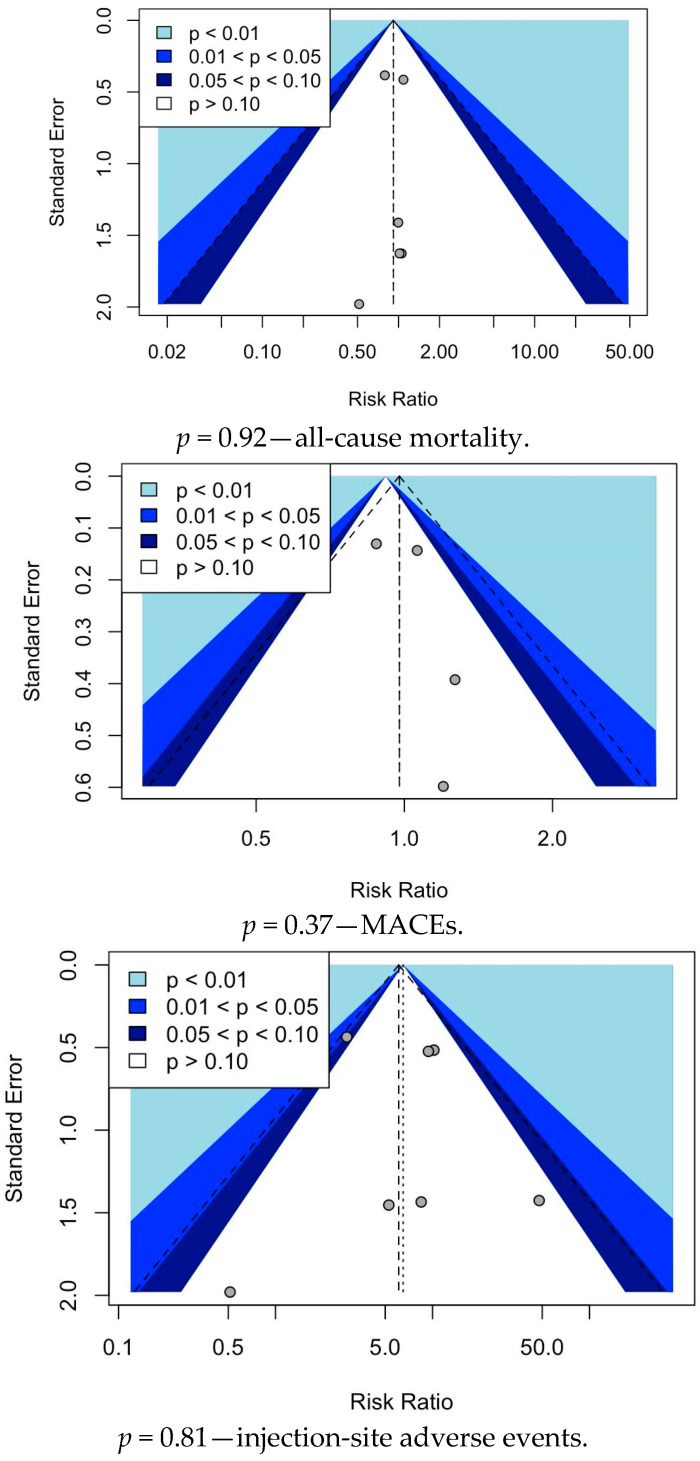

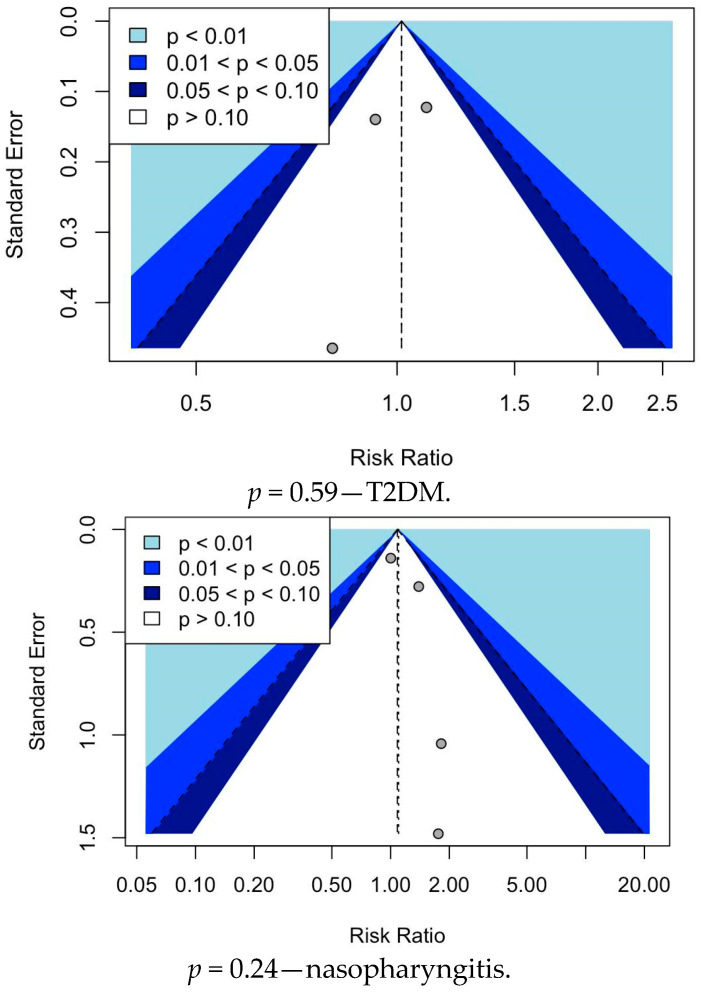
Funnel plot analysis.

Outcomes were assessed using relative risks (RRs) obtained from each trial. Random-effects meta-analyses were conducted using the Mantel–Haenszel method for study weighting and using the restricted maximum likelihood (REML) method as a heterogeneity estimator because of the between-trial variance [27]. Effect estimates were presented with a 95% confidence interval (CI) [27]. Heterogeneity was assessed using I^2^ statistics, proposed by Higgins and Thompson, with estimated values of I^2^ < 25%, 25% < I^2^ < 50%, and I^2^ > 50% indicating low, moderate, and high heterogeneity, respectively [28]. The subgroup analysis was performed based on single and multiple dosing regimens. The funnel plots for each outcome are in Figure 2. RStudio with the “meta” package (eMethod) was used to perform the entire statistical analysis [29].

### 2.4. Quality Assessment

The quality assessment of included studies was conducted using the revised Cochrane risk-of-bias tool for randomized trials (Rob2) [30]. The quality assessment found all seven studies demonstrated an overall low risk of bias, as shown in Supplementary Table S1.

## 3. Results

### 3.1. Study Selection and Characteristics

The preliminary database search yielded 218 potentially eligible articles. After a meticulous search and application of the predetermined inclusion criteria, six articles containing seven RCTs were included in the final meta-analysis. This yielded a total population of 4790 patients with 1591 (33.2%) females and a mean age of 63.8 ± 9.7 years. Then, 2416 out of 4790 patients were administered Inclisiran in either a single-dose or multiple-dose regimen. The mean weighted follow-up duration was 15.8 months or 475 days [15,19,20,21,22,25]. Most patients had a profile of ASCVD with at least low-density lipoprotein (LDL) > 70 mg/dL on maximally tolerated statins with no use of PCKS9 monoclonal antibodies within the last 90 days of screening [14,18,19,20,21,24]. Adequate randomization in all seven RCTs minimized the heterogeneity between the treatment and control groups. Details of included studies and patient characteristics are described in Table 1, Table 2, and Table 3, respectively.

### 3.2. Outcomes

**Figure 3:**
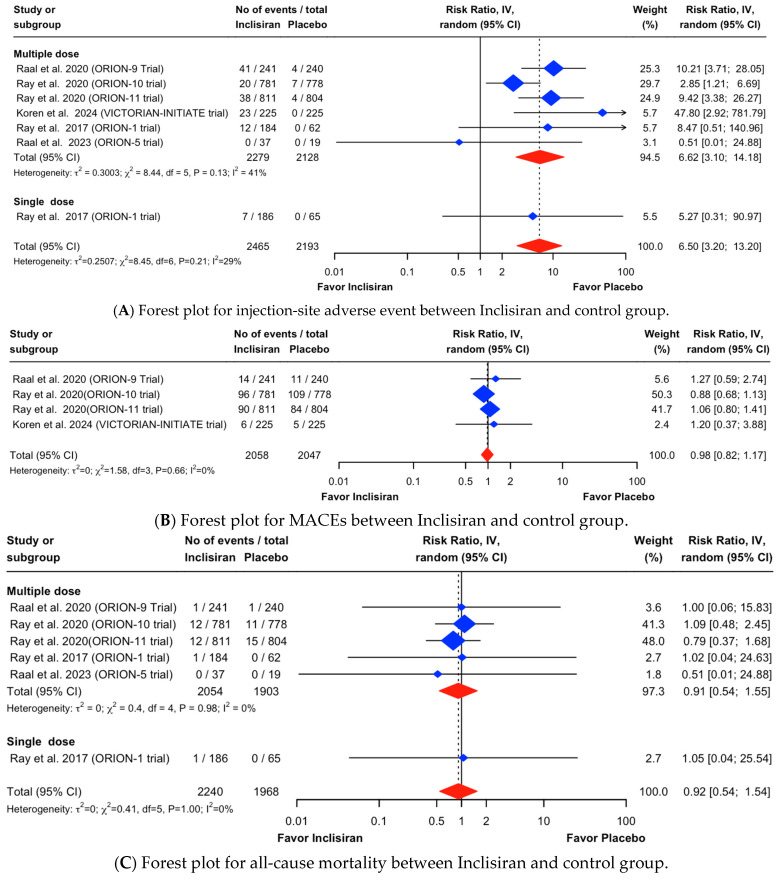

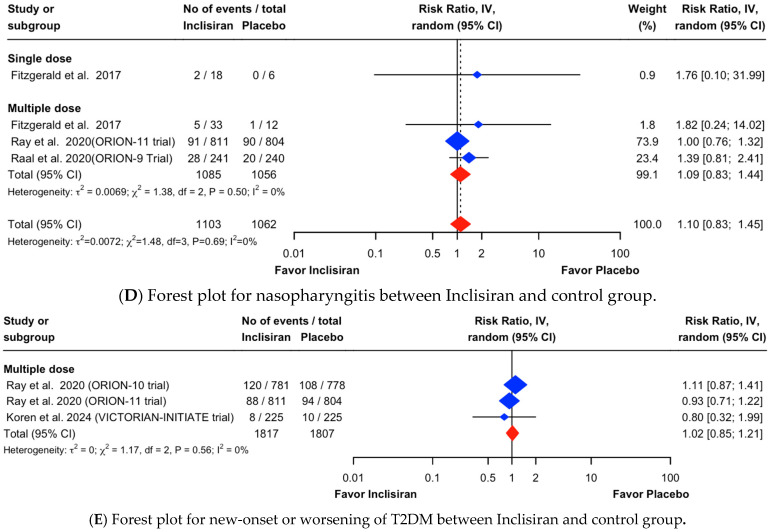
Forest plot for (**A**) injection-site adverse event, (**B**) MACEs, (**C**) all-cause mortality, (**D**) nasopharyngitis, and (**E**) new-onset or worsening T2DM between Inclisiran and control group [14,18,19,20,21,24].

The outcomes of interest were all-cause mortality, MACEs, injection-site adverse events, new-onset or worsening T2DM, and nasopharyngitis in patients who were administered Inclisiran. The use of Inclisiran caused a significant increase in injection-site adverse events (RR, 6.50; 95% CI, 3.20 to 13.20; I^2^ = 29%). However, there were no statistically significant differences in MACEs (RR, 0.98; 95% CI, 0.82 to 1.17; I^2^ = 0%), all-cause mortality (RR, 0.92; 95% CI, 0.54 to 1.54; I^2^ = 0%), nasopharyngitis (RR, 1.10; 95% CI, 0.83 to 1.45; I^2^ = 0%), and T2DM (RR, 1.02; 95% CI, 0.85 to 1.21; I^2^ = 0%). The results of all outcomes are shown in the forest plot in Figure 3.

## 4. Discussion

In this systematic review and meta-analysis of RCTs comprising 4790 patients, demonstrating the safety of the use of Inclisiran in patients with dyslipidemia, we found a higher incidence of injection-site-related adverse events in patients receiving Inclisiran. However, Inclisiran did not increase all-cause mortality or MACEs. Furthermore, the risk of other side effects, like nasopharyngitis and new-onset or worsening T2DM, was not significantly higher in patients treated with Inclisiran.

Inclisiran’s safety profile is clinically significant due to its novel mechanism of action and unique biannual dosing regimen, which enhances patient convenience and adherence compared to daily statin or biweekly PCSK9 monoclonal antibody used. Its low incidence of TEAEs and demonstrated efficacy in reducing LDL-C levels position it as a valuable option for patients who are statin-intolerant, unable to achieve target LDL-C levels with existing therapies, or at high ASCVD risk. Additionally, its compatibility with other lipid-lowering agents provides flexibility in individualized treatment plans. While its long-term safety and cardiovascular benefits require further study, Inclisiran offers a promising alternative for improving lipid management and addressing adherence challenges in dyslipidemia care.

### 4.1. Mortality and Cardiovascular Outcomes

Mortality and MACEs are the key outcomes that are studied in any long-term drug-based cardiovascular trials. Our findings showed no significant change in the risk of all-cause mortality (RR 0.92, 95% CI 0.54–1.54) or MACEs (RR 0.98, 95% CI 0.82–1.17) with Inclisiran use. This is crucial as it reinforces the safety of Inclisiran in reducing LDL cholesterol without elevating cardiovascular risk, which remains an important consideration in the context of the drug’s long-term use in patients with chronic dyslipidemia. However, our findings differ from those of Asbeutah et al. and Cicero et al., who reported a reduction in MACEs [31,32]. Both studies, however, acknowledged that estimating the relative risk reduction in MACEs with Inclisiran is challenging due to the available short-term data, and they included fewer RCTs than our present study [31,33].

The differences in findings may be due to the smaller number of RCTs used by Asbeutah et al. and Cicero et al., which could have been influenced by limited data or shorter observation windows [31,33]. Variations in patient baseline characteristics, such as cardiovascular risk or comorbidities, may also contribute to these discrepancies. These differences are important for clinical decision-making, as our study suggests that Inclisiran does not increase cardiovascular risk, while Asbeutah et al. and Cicero et al. indicate potential benefits in reducing MACEs, particularly in high-risk populations [31,32]. This underscores the need for further research to clarify the long-term effects of Inclisiran on mortality and MACEs, especially in diverse groups with varying cardiovascular risks, and highlights the importance of considering both lipid-lowering efficacy and the broader cardiovascular impact when assessing new dyslipidemia treatments, especially in patients with chronic or complex conditions.

### 4.2. Impact on T2DM

Another important finding was the absence of an increased risk of new-onset or worsening T2DM (RR 1.02, 95% CI 0.85–1.21), which is a common side effect associated with statin therapy. Statins have been linked to insulin resistance and the subsequent development of diabetes; however, Inclisiran’s targeted mechanism offers a promising advantage by focusing on PCSK9, thereby bypassing multiple metabolic pathways and suggesting a lower risk of interference with glucose metabolism [16,17,32]. This positions Inclisiran as a safer alternative for patients with or at risk of diabetes, a population that often requires stringent cardiovascular management.

### 4.3. Respiratory and Immune Effects

We also found that Inclisiran did not increase the risk of nasopharyngitis (RR 1.10, 95% CI 0.83–1.45), which was one of the most frequently reported complications, occurring in more than 3% of the population. In comparison, previous studies, such as those analyzed by Cicero et al., noted a slight increase in respiratory complications such as bronchitis with Inclisiran [33]. The proposed mechanism for respiratory tract infections associated with Inclisiran is mast cell degranulation in the airways [33]. However, recent studies indicate that Inclisiran does not have a significant effect on immune cells, including leukocytes, monocytes, and neutrophils, as well as inflammatory biomarkers [34]. Conversely, Inclisiran has been shown to reduce immune cascade by reducing Toll-like receptor 4 levels in circulating monocytes [32,35]. Our findings align with the latter outcome, demonstrating that Inclisiran does not significantly increase the incidence of upper respiratory tract infections in immunological pathways.

The clinical implications of these differences are important, emphasizing the complexity of assessing Inclisiran’s safety in various patient populations and conditions. While our study indicates that Inclisiran does not significantly increase the risk of respiratory infections via immunological mechanisms, Cicero et al.’s study suggests a potential slight increase in respiratory complications in certain individuals, which warrants further exploration [33]. Clinicians should carefully consider patient factors, such as underlying immune conditions, when prescribing Inclisiran. These findings highlight the need for more comprehensive and long-term studies to fully evaluate Inclisiran’s immunological effects, especially in diverse populations with varying health profiles and risk factors.

#### Incidence of Injection-Site Adverse Events

Our analysis revealed an increased relative risk of 6.50 (95% CI, 3.20 to 13.20) for injection-site adverse events. These reactions were generally mild to moderate in severity, with most resolving without the need for medical intervention [14,18,19,20,21,24]. Commonly reported injection-site reactions included localized erythema, swelling, pain, and pruritus, with occasional occurrences of rash and thrombophlebitis [14,18,19,20,21,24]. These reactions were transient and rarely resulted in treatment discontinuation [14,18,19,20,21,24].

Fitzgerald et al. noted no injection-site-related adverse effects with Inclisiran, likely due to their lower initial doses of 25 mg and shorter follow-up period of 56 days compared to other trials such as ORION-9, ORION-10, and ORION-11, which involved higher doses and longer durations [14,20,21]. For instance, the ORION-9 trial, which employed a higher initial dose of 300 mg and had a longer follow-up period of 450 days, reported injection-site reactions in 17% of patients treated with Inclisiran compared to a rate of 1.7% in the placebo group [21]. These reactions were mild, non-persistent, and without any significant impact on the overall safety profile of the drug [21]. Differences in complication rates may arise from these varying dosing schedules and the characteristics of study populations, including underlying health conditions and treatment duration. Given that Inclisiran is administered subcutaneously, localized irritation may be related to the drug’s formulation or delivery method. Subcutaneous injections can cause tissue irritation, particularly when higher volumes or concentrations of the drug are used, which could account for the recurring nature of these reactions. This phenomenon is not exclusive to Inclisiran, as it is also observed with other injectable biologics. Therefore, future studies should explore whether modifications in formulation or administration techniques could alleviate these side effects, as they are crucial for patient comfort and long-term adherence.

The mild injection-site reactions and absence of significant immunological side effects observed in Inclisiran studies can be attributed to several underlying mechanisms. Subcutaneous administration, while effective for delivering therapeutic agents, can cause localized irritation due to the physical presence of the injection or the properties of the drug formulation. For Inclisiran, these mild reactions—such as erythema, swelling, and pruritus—are likely a result of transient immune responses to the injection process rather than the drug itself, a phenomenon commonly observed with injectable biologics [14,18,19,20,21,24]. Such reactions are transient, rarely persistent, and generally do not necessitate medical intervention or treatment discontinuation [14,21].

The absence of significant systemic immunological side effects suggests that Inclisiran has a low potential for inducing broader immune activation. Inclisiran’s targeted mechanism of action—silencing PCSK9 gene expression via RNA interference—limits systemic exposure to antigenic protein products, thereby reducing the likelihood of immune-mediated adverse events [14,21]. The chemical modifications in Inclisiran, including stabilization with 2′-O-methyl groups and phosphorothioate linkages, enhance the molecule’s stability and reduce recognition by the immune system, further minimizing off-target effects and systemic immunological responses [24].

These mechanisms collectively contribute to the favorable safety profile observed in clinical trials, making Inclisiran a promising therapeutic option for the long-term management of ASCVD. Future studies exploring formulation refinements or alternative delivery methods could further mitigate injection-site reactions and enhance patient comfort, ensuring better patient adherence to therapy [14,18,19,20,21,24].

The mild and transient nature of injection-site reactions, such as localized erythema, swelling, pain, and pruritus, underscores their limited clinical relevance, particularly given their self-resolving nature and minimal impact on treatment adherence. These reactions rarely lead to discontinuation and pose no significant safety concerns, making them manageable with patient education and reassurance. Their predictability highlights the strong overall safety profile of the drug, particularly in the context of long-term therapies where tolerability is critical for adherence. By framing these events as expected and minor, clinicians can mitigate concerns and support positive treatment outcomes.

## 5. Strengths and Limitations

Our study has several notable strengths. It significantly expands upon the findings of previous meta-analyses, such as Cicero et al., which included only five RCTs and assessed fewer safety endpoints [33]. In contrast, our study incorporated data from seven RCTs and found no significant change in MACEs and nasopharyngitis, differing from Cicero et al., which reported a significant reduction in MACEs and an increase in respiratory tract infections with Inclisiran [33]. Additionally, our findings exhibit minimal heterogeneity, further reinforcing the robustness of our safety assessments. This low heterogeneity across studies, despite varying dosing regimens and populations, may result from Inclisiran’s robust and uniform biological effect, which remains effective across different conditions. Additionally, variations in dosing might still fall within a therapeutic range where efficacy remains consistent. Standardized study designs and similar inclusion criteria across populations further minimize variability, leading to consistent findings across studies. Therefore, our study provides a more extensive review of RCTs evaluating the safety of Inclisiran in patients with dyslipidemia.

However, the trials in our study are phase 2 or 3, with a relatively short follow-up period (mean ~15.8 months), which limits the ability to comprehensively evaluate long-term safety outcomes, particularly in the context of chronic diseases like ASCVD. Managing ASCVD often requires lifelong therapy, and potential cumulative effects or rare adverse events may only become apparent over extended periods. Additionally, the studies evaluated various drug regimens, and this was not a pooled patient-level meta-analysis. However, our findings closely align with those of Wright et al., who conducted a patient-level meta-analysis of the ORION-9, ORION-10, and ORION-11 trials [36]. Their analysis showed that injection-site adverse events were more common with Inclisiran compared to a placebo (5.0% vs. 0.7%), but these events were predominantly mild, with none being severe or persistent [36]. This consistency reinforces the validity of our the findings ot our meta-analysis. Despite these limitations, this is the most comprehensive analysis of RCTs examining the safety of Inclisiran in patients with dyslipidemia and ASCVD or risk equivalents.

## 6. Future Directions and Pharmacovigilance

### 6.1. Future Directions

To build upon the findings of our study and address existing gaps, future long-term studies of Inclisiran should focus on longer follow-up periods (5–10 years) to capture potential delayed adverse events and assess sustained outcomes. These studies should utilize patient-level meta-analyses to account for individual risk factors and heterogeneity in treatment effects. Standardized endpoints for adverse events, particularly respiratory complications and MACEs, should be established to ensure consistency across trials. In-depth subgroup analyses based on age, sex, comorbidities, and prior therapies would help to identify specific patient populations who may experience different safety profiles or benefit from Inclisiran. Additionally, head-to-head comparisons with other lipid-lowering agents like statins and PCSK9 inhibitors are necessary to understand Inclisiran’s relative benefit–risk profile better.

Furthermore, future research should monitor immune responses and inflammatory markers to evaluate any immunological mechanisms involved in respiratory tract infections or other adverse events. Incorporating real-world data through registries and observational studies will provide insights into Inclisiran’s performance in diverse populations outside of clinical trial settings. Finally, long-term cardiovascular outcomes and mortality should remain key focus areas, with studies designed to assess whether Inclisiran offers sustained reductions in MACEs and mortality, particularly in high-risk groups such as those with familial hypercholesterolemia or severe ASCVD. These methodologies will help to provide a comprehensive understanding of Inclisiran’s safety and efficacy in the long term.

### 6.2. Pharmacovigilance

To enhance the safety of Inclisiran and increase adverse drug reaction (ADR) reporting, it is essential to implement robust pharmacovigilance strategies. Simplifying reporting systems, such as integrating ADR reporting into electronic health records (EHRs) or using user-friendly mobile applications, can encourage healthcare professionals (HCPs) to report ADRs promptly [37]. Educational initiatives targeting HCPs to raise awareness about pharmacovigilance and the importance of reporting, combined with patient education on identifying and reporting side effects, can improve reporting rates [38]. Providing feedback to reporters about their submissions and leveraging incentives or recognitions can further motivate participation [39]. For Inclisiran, these strategies, alongside regulatory mandates for monitoring long-term safety in real-world settings, can enhance ADR detection and contribute to patient safety by addressing potential risks systematically.

## 7. Conclusions

Our meta-analysis indicates that Inclisiran has an acceptable overall safety profile, although it is associated with an increase in injection-site adverse effects. Due to its favorable effect on dyslipidemia in high-risk individuals with ASCVD or risk equivalents, this safety profile highlights Inclisiran’s potential role in comprehensive lipid management. However, the relatively short follow-up durations in the included trials pose important implications for the generalizability of findings to long-term use of Inclisiran. While these trials demonstrate significant reductions in LDL-C levels and favorable short-term safety profiles, the limited follow-up periods may not capture potential long-term adverse effects or the durability of efficacy. This constraint is particularly critical for therapies like Inclisiran, which are intended for chronic use in managing dyslipidemia and reducing ASCVD risk. As such, the absence of extended observational data may lead to an incomplete understanding of long-term safety, adherence patterns, and sustained clinical benefits. Further, longer-term follow-ups and well-designed prospective cohort studies, such as ORION-4 and VICTORIAN-2P/1P, will provide additional insights into Inclisiran’s utility and safety profile, particularly regarding MACEs and injection-site reactions in the management of dyslipidemia [40]. These studies will help to establish clear guidelines for the optimal use of Inclisiran as a lipid-lowering agent.

## Figures and Tables

**Table 1:** Study characteristics.

Study	Country	Design	Regimen of Inclisiran Administration	Population with Inclisiran Administration (*n*)	Control (*n*)	Dose of Inclisiran Administered	Type of Control	Follow-Up Duration (Days)
Ray et al., 2017 (ORION-1 trial) [18]	Canada, Europe, and the United States	Randomized, multicenter, double-blind, placebo-controlled, phase 2 clinical trial	Single dose	186	65	A single dose, 200 mg, 300 mg, or 500 mg, of Inclisiran on day 1	Placebo	On days 14, 30, 60, 90 (end-of-treatment visit), 120, 150, 180, 210 (last trial visit)
			Multiple doses	184	62	Two doses of 100 mg, 200 mg, or 300 mg of Inclisiran on day 1 and day 90	Placebo	
Fitzgerald et al., 2017 [14]	United Kingdom	Randomized, multicenter, single-blind, placebo-controlled, phase 1 clinical trial	Single dose	18	6	A single dose of 25 mg, 100 mg, 300 mg, 500 mg, or 800 mg of Inclisiran on day 1	Placebo	On day 56 (last day of trial)
			Multiple doses	33	12	300 mg or 500 mg two-monthly doses or 125 mg weekly for four doses, 250 mg every other week for two doses	Placebo	On days 56 and 180 (last day of trial)
Raal et al., 2020 (ORION-9 trial) [21]	South Africa, Switzerland, England, USA, Germany, Canada	Randomized, multicenter, double-blind, placebo-controlled, phase 3 clinical trial	Multiple doses	242	240	Four doses of 300 mg of Inclisiran administered on days 1, 90, 270, and 450	Placebo	On days 30, 150, 330, 510 (end-of-treatment visit), 540 (last day of trial)
Ray et al., 2020 (ORION-10 trial) [20]	USA	Randomized, multicenter, double-blind, placebo-controlled, phase 3 clinical trial	Multiple doses	781	780	Four doses of 300 mg Inclisiran administered on days 1, 90, 270, and 450	Placebo	On days 30, 150, 330, 510 (end-of-treatment visit), 540 (last day of trial)
Ray et al., 2020 (ORION-11 trial) [20]	Europe and South Africa	Randomized, multicenter, double-blind, placebo-controlled, phase 3 clinical trial	Multiple doses	810	807	Four doses of 300 mg Inclisiran administered on days 1, 90, 270, and 450	Placebo	On days 30, 150, 330, 510 (end-of-treatment visit), 540 (last day of trial)
Raal et al., 2023 (ORION-5 trial) [19]	Hong Kong, Israel, Russia, Serbia, South Africa, Taiw- an, Turkey, and Ukraine	Randomized, multicenter, double-blind, placebo-controlled, phase 3 clinical trial with 2 parts in which only part 1 is placebo-controlled	Multiple doses	37	19	Two doses of 300 mg of Inclisiran on day 1 and day 90	Placebo	On day 180 (last day of trial-part 1)
Koren et al., 2024 (VICTORIAN-INITIATE trial) [24]	USA	Randomized, multicenter, open-label, Phase 3b trial	Multiple dose	225	225	Three doses of 300 mg Inclisiran administered on days 0, 90, and 270	Usual care which consists of lipid management without Inclisiran, at treating physician’s discretion	On days 0, 90, 180, 270 (end-of-treatment visit) and 330 (last day of trial)

Abbreviations: RCT: randomized controlled trial. Summary: The phase 1 to phase 3 trials, including ORION-1, ORION-5, ORION-9, ORION-10, ORION-11, and VICTORIAN-INITIATE, conducted globally, demonstrated Inclisiran’s efficacy in significantly reducing LDL cholesterol with a favorable safety profile across single- and multiple-dose regimens over follow-up periods of 56 to 540 days.

**Table 2:** Strengths and weaknesses of included trials.

Study (Trial)	Strengths	Weaknesses
**Ray et al., 2017** (**ORION-1**) [18]	- Phase 2, multicenter, double-blind design, ensuring rigorous evaluation.	- Limited follow-up duration (up to 210 days) restricts understanding of long-term safety and efficacy.
	- Includes both single- and multiple-dose regimens, providing comparative insights.	- Relatively small sample size for subgroup analyses, particularly in single-dose groups.
**Fitzgerald et al., 2017 [14]**	- Phase 1 trial with dose-ranging assessments, providing foundational pharmacokinetics data.	- Small sample size and single-blind design may limit generalizability and introduce bias.
	- Assessed multiple dosing regimens, including weekly and biweekly schedules.	- Very short follow-up periods (56 to 180 days) do not capture long-term outcomes.
**Raal et al., 2020** (**ORION-9**) [21]	- Large, phase 3, multicenter, double-blind trial with robust control design.	- Excluded patients with moderate to severe hepatic or renal impairment, limiting generalizability to high-risk populations.
	- Focused on a high-risk heterozygous familial hypercholesterolemia population, addressing an underserved group.	- The follow-up duration of 540 days may not fully evaluate long-term adverse events or durability of efficacy.
**Ray et al., 2020** (**ORION-10**) [20]	- Phase 3, double-blind design with a large population enhances the reliability of results.	- Follow-up limited to 540 days, with no extension phase to assess prolonged safety and efficacy.
	- Evaluated standard 300 mg dose over multiple administrations, aligning with clinical practice.	- Geographic focus on the USA limits the diversity of patient demographics compared to other global trials.
**Ray et al., 2020** (**ORION-11**) [20]	- Included a diverse population from Europe and South Africa, improving generalizability.	- Similar follow-up limitation as ORION-10, with no data on outcomes beyond 540 days.
**Raal et al., 2023** (**ORION-5**) [19]	- Conducted in multiple countries, representing a diverse patient demographic.	- Part 1 of the study limited to a placebo-controlled design, with no long-term data from part 2 included in the analysis.
	- Focused on patients with homozygous familial hypercholesterolemia, addressing a high-risk group.	- Small sample size limits the power of statistical analyses and subgroup evaluations.
**Koren et al., 2024** (**VICTORIAN-INITIATE**) [24]	- Open-label phase 3b trial provides real-world insights into Inclisiran use with usual care as a comparator.	- Open-label design introduces potential for performance and detection bias.
	- Follow-up duration (330 days) shorter than other phase 3 trials.	- Usual care comparator does not ensure consistency in control treatment, introducing variability in the results.

**Table 3:** Baseline characteristics of patients.

	Ray et al. (ORION-1 Trial) [18]				Fitzgerald et al. [14]				Raal et al. (ORION-5 Trial) [19]		Raal et al. (ORION-9 Trial) [21]		Ray et al. (ORION-10 Trial) [20]		Ray et al. (ORION-11 Trial) [20]		Koren et al. (VICTORIAN-INITIATE Trial) [24]	
	Single-Dose Regimen		Multiple-Dose Regimen		Single-Dose Regimen		Multiple-Dose Regimen		Multiple-Dose Regimen		Multiple-Dose Regimen		Multiple-Dose Regimen		Multiple-Dose Regimen		Multiple-Dose Regimen	
	Inclisiran (%)	Control (%)	Inclisiran (%)	Control (%)	Inclisiran (%)	Control (%)	Inclisiran (%)	Control (%)	Inclisiran (%)	Control (%)	Inclisiran (%)	Control (%)	Inclisiran (%)	Control (%)	Inclisiran (%)	Control (%)	Inclisiran (%)	Control (%)
Age (years ± SD/IQR)	63.3 ± 12.1	62.0 ± 11.4	63.9 ± 9.9	62.8 ± 10.3	46 ± 10	48 ± 14	51.8 ± 13	53.3 ± 11.8	43.8 ± 13.4	40.7 ± 12.1	56 (47–63)	56 (46–64)	64.8 ± 8.3	64.8 ± 8.7	63.3 ± 12.1	62.0 ± 11.4	66 (35–87)	68 (27–89)
Female	32.3	35	33.7	47	6	67	42.5	37.5	62.2	57.9	53.7	52.1	31.5	29.7	28.5	28	29.8	32
White	91.7	92	94.7	94	67	67	73	94	NA	NA	93.4	94.6	83.6	87.8	97.7	98.6	82.7	83.1
ASCVD	68	69	68	74	NA	NA	NA	NA	67.6	68.4	24.4	30.4	100	100	87.9	87	93.8	96.9
ASCVD risk equivalent	NA	NA	NA	NA	NA	NA	NA	NA	32.4	31.6	NA	NA	0	0	12.1	13	NA	NA
Current smoker	NA	NA	NA	NA	NA	NA	NA	NA	10.8	10.5	11.6	11.7	15.7	14.2	19.8	16.4	NA	NA
HTN	NA	NA	NA	NA	NA	NA	NA	NA	40.5	31.6	42.1	42.1	91.4	89.9	79	81.9	40.5	31.6
DM	NA	NA	NA	NA	NA	NA	NA	NA	5.4	5.3	8.3	11.7	47.5	42.4	36.5	33.7	42.2	42.2
Familial hypercholesterolemia	NA	NA	NA	NA	NA	NA	NA	NA	100	100	NA	NA	1	1.5	1.7	1.7	NA	NA
Total cholesterol (mg/dL ± SD)	206.9 ± 50.5	207.7 ± 59	216.2 ±71.7	208.4 ± 54.7	NA	NA	NA	NA	363.0 ± 131.8	423.2 ± 122.4	230 ± 54.6	232.4 ± 62.8	180.6 ± 46.1	180.6 ± 43.6	187.3 ± 48.2	183.3 ± 42.8	171.8 ± 40.1	171.7 ± 37.1
LDL (mg/dL ± SD)	126.1 ± 41.5	128.5 ± 51.3	132.9 ± 63.1	125.2 ± 44.3	163 ± 32.9	131.5 ± 19.3	140.4 ± 31.2	135.4 ± 50	294.0 ± 136.3	356.7 ± 122.4	151.4 ± 15.1	154.7 ± 58.0	104.5 ± 39.6	104.8 ± 37.0	107.2 ± 41.8	103.7 ± 36.4	97.4 ± 33.2	97.4, 32 ± 4
HDL (mg/dL ± SD	50 ± 13.6	49.9 ± 13.6	47.6 ± 13.5	51.2 ± 16.1	NA	NA	NA	NA	NA	NA	51.5 ± 15.1	50.8 ± 13.1	46.6 ± 14.3	45.9 ± 14.4	49.7 ± 15.5	49.3 ± 13.8	44.8 ± 10.8	47.7 ± 13.4
Non-HDL (mg/dL ± SD)	156.8 ± 49.8	157.8 ± 55.2	165.6 ± 70.6	157.1 ± 53.7	NA	NA	NA	NA	317.6 ± 136.8	380.7 ± 124.5	178.5 ± 55.4	181.5 ± 62.5	134.0 ± 44.5	134.7 ± 43.5	137.6 ± 46.9	133.9 ± 41	127.4 ± 39.4	124 ± 36.1
LpA (mg/dL)/(median and IQR)	1.9 (0.7–7.7)	1.5 (0.4–6.7)	2.2 (0.6–7.9)	2.8 (0.4–8.5)	NA	NA	NA	NA	3.9 (1.2–9)	5.1 (1.7–7.5)	3.1 (1.2–9.9)	3.0 (1.1–10.2)	3.1 (1.0–10.0)	3.1 (1.1–10.4)	2.3 (1.0–9.8)	1.9 (1.0–10.0)	20.5 (8–58.7)	19.8 (8–68.7)
ApoB (mg/dL ± SD)	103.4 ± 27	102.4 ± 29.6	107.8 ± 38.2	104.6 ± 31.5	NA	NA	NA	NA	192.7 ± 71.8	223.4 ± 70.2	123.8 ± 33.2	124.5 ± 34.8	94.1 ± 25.6	94.6 ± 25.1	97.1 ± 28	95.1 ± 5.2	94 ± 253	90.9 ± 24.4
Triglycerides (mg/dL ± SD)/(median and IQR)	126.3 (90–173.7)	125 (95–170)	128.3 (95.3–194.3)	137 (103–187)	135.5 ± 55.7	70.9 ± 12.4	121.5 ± 77.4	132.9 ± 41.2	NA	NA	120 (82–167)	119 (85–166)	127 (92–181)	129 (96–182)	135 (99–181)	135 (102–185)	132 (95–187)	119 (87–170)
PCKS9 (μg/L ± SD)	428.2 ± 135.7	404.7 ± 131.3	416.1 ± 133.3	431.3 ± 132.3	275.4 ± 58.2	279 ± 99.5	353.8 ± 106.2	337.7 ± 106.4	606.1 ± 447	499.2 ± 211	452.2 ± 131.2	429.1 ± 135.3	422.1 ± 176.9	414.9 ± 145.7	355 ± 98.9	353 ± 97.4	NA	NA
Statin use	74.3	70	70.3	77	NA	NA	27.27	33.3	NA	NA	90.5	90.4	89.8	88.7	94.6	94.9	89.8	88
High-intensity statin use	NA	NA	NA	NA	NA	NA	NA	NA	100	100	76.4	71.2	67.2	68.8	79	78.2	NA	NA
Ezetimibe use	NA	NA	NA	NA	NA	NA	NA	NA	63.2	67.6	55.8	50	10.2	9.5	6.3	7.7	NA	NA

Notes: %—percentage; SD—standard deviation; IQR—interquartile range; NA—not applicable; mg/dL—milligrams/deciliter; μg/L—microgram/liter. Abbreviations: ASCVD: atherosclerotic cardiovascular disease; HTN: hypertension; DM: diabetes mellitus; LDL: low-density lipoprotein; HDL: high-density lipoprotein; LpA—lipoprotein A; ApoB—apolipoprotein B; PCKS9—proprotein convertase subtilisin/kexin type 9. Summary: Inclisiran was evaluated in several clinical trials across diverse populations, including ORION-1, ORION-5, ORION-9, ORION-10, ORION-11, and VICTORIAN-INITIATE, showing up to a 50% reduction in LDL cholesterol and significant decreases in PCSK9 levels. The trials, involving participants on high-intensity statin therapy (67–94%) and some on ezetimibe, demonstrated consistent efficacy across subgroups based on age, sex, and cardiovascular risk, with a favorable safety profile and low adverse event rates. Inclisiran also proved effective in patients with familial hypercholesterolemia, high cardiovascular risk, and those with or without diabetes or hypertension.

## Supplementary Materials

The following supporting information can be downloaded at: https://www.mdpi.com/article/10.3390/healthcare13020141/s1. Table S1: Summary table of results from RoB 2 bias analysis of RCTs.
