# Supplementary material for: The Safety Profile of Inclisiran in Patients with Dyslipidemia: A Systematic Review and Meta-Analysis
Source: Healthcare (Basel). 2025 Jan 13;13(2):141. doi: 10.3390/healthcare13020141 (PMC11764983; doi:10.3390/healthcare13020141)
Supplement: Supplementary file 1 [file healthcare-13-00141-s001.zip › healthcare-3351035-supplementary.pdf]

**Study**	**Risk of bias domains**					
	**D1**	**D2**	**D3**	**D4**	**D5**	**Overall**
Koren et al. 2024 (VICTORIAN-INITIATE)	+	+	+	+	+	+
Raal et al. 2020 (ORION-5)	+	+	−	+	+	+
Raal et al. 2020 (ORION-9)	+	+	+	−	+	+
Ray et al. 2020 (ORION-10)	+	+	+	−	+	+
Ray et al. 2020 (ORION-11)	+	+	+	−	+	+
Fitzgerald et al. 2017	+	+	+	+	+	+
Ray et al. 2017(ORION-1)	+	+	+	+	+	+

Domains:
D1-Bias arising from the randomization process
D2-Bias due to deviations from intended interventions
D3-Bias due to missing outcome data
D4-Bias in measurement of the outcome
D5-Bias in selection of the reported result

Judgement

+ Low risk of bias

− Some concerns of bias

X High risk of bias

Supplemental Table S1: Summary table of results from RoB 2 bias analysis of RCTs
